# Ability-Related Emotional Intelligence: An Introduction

**DOI:** 10.3390/jintelligence12050051

**Published:** 2024-05-19

**Authors:** Michael D. Robinson

**Affiliations:** Psychology, NDSU Department 2765, P.O. Box 6050, Fargo, ND 58108-6050, USA; michael.d.robinson@ndsu.edu

**Keywords:** emotional intelligence, ability, emotion, measurement, theory

## Abstract

Emotionally intelligent people are thought to be more skilled in recognizing, thinking about, using, and regulating emotions. This construct has garnered considerable interest, but initial enthusiasm has faded and it is time to take stock. There is consensus that ability-related measures of emotional intelligence (EI) can be favored to self-report tests, in part because the resulting scores cannot be equated with personality traits. However, there are questions surrounding measurement as well as predictive value. Experts in the field were encouraged to chart new directions, with the idea that these new directions could reinvigorate EI scholarship. Special Issue papers speak to theory, mechanism, measurement, and training. In addition, these papers seek to forge links with research traditions focused on interpersonal perception, emotional awareness, and emotion regulation. As a result of these efforts, new insights into what EI is and how it works can be anticipated in upcoming years.

## 1. Special Issue on Ability-Related Emotional Intelligence: An Introduction

Emotions figure prominently in many realms such as decision making (Lerner et al. 2015), relationships (Engelberg and Sjöberg 2004), and well-being (Watson 2000). Owing to such links, emotion-related capacities might be expected to help individuals succeed rather than fail as they negotiate the complexities of daily life. Modern interest in emotional intelligence (EI)—which is thought to encompass skills related to the identification, understanding, management, and use of emotions (Kotsou et al. 2019)—began with a definitional effort by Salovey and Mayer (1990). Goleman (1995) then popularized the construct by arguing, without sufficient evidence, that EI could be more important than general mental ability in determining whether lives were successful or not. These popularization efforts, which culminated in a Times magazine piece and an Oprah Winfrey episode (Roberts et al. 2010), inevitably led some to suggest that interest in EI could be likened to a fad that would surely perish, like the dodo bird (Antonakis et al. 2009). This has not happened, but there are major questions concerning the construct as well as its value in predicting real-world outcomes (Zeidner et al. 2008).

It is often suggested that there is confusion about whether emotional intelligence should be thought of in terms of personality traits, which can be self-reported, or abilities, which require performance-based tests (Matthews et al. 2004). Although it is useful to compare the predictive validities of these two types of tests (MacCann et al. 2020a; Martins et al. 2010), there seems to be enough consensus to state that these two modes of assessment need to be distinguished from each other, in part because self-reports of EI rarely correlate highly with ability-based assessments of EI (Roberts et al. 2010). In many cases, self-reports of EI display greater predictive validity (e.g., Martins et al. 2010), but such tests also correlate highly with standard personality trait measures, rendering their discriminant validity suspect (Joseph et al. 2015). And, if one endorses an ability-based perspective on EI, which one arguably should (Mayer et al. 2008), the field will need to prioritize the ability-based model and its assessments (Daus and Ashkanasy 2005; Roberts et al. 2010). The current Special Issue does so.

Ability-based tests seek to determine whether individuals are good at perceiving emotions, whether they understand how emotions work, and whether they can (at least as inferred from their responses to standardized test materials) manage emotions in effective ways (Joseph and Newman 2010). These tests, more or less, assess emotion-related knowledge and its application (Hoemann et al. 2021) and they seek to place individuals along a continuum, from low to medium to high levels of emotion-related ability (Joseph and Newman 2010). The first widely used test was the Multifactor Emotional Intelligence Scale (MEIS: Mayer et al. 1999). This test was followed by the Mayer–Salovey–Caruso Emotional Intelligence Test (MSCEIT: Mayer et al. 2003), the Situational Test of Emotional Understanding (STEU: MacCann and Roberts 2008), the Situational Test of Emotion Management (STEM: MacCann and Roberts 2008), the North Dakota Emotional Abilities Test (NEAT: Krishnakumar et al. 2016), and the Geneva Emotional Competence Test (GECo: Schlegel and Mortillaro 2019). Assessment-inclined researchers have also developed a number of tests of emotion recognition ability, which will relate to the perception branch of EI (Mayer et al. 2003), including the Matsumoto and Ekman’s Japanese and Caucasian Brief Affect Recognition Test (JACBART: Matsumoto et al. 2000), the Multimodal Emotion Recognition Test (MERT: Bänziger et al. 2009), and the Geneva Emotion Recognition Test (GERT: Schlegel et al. 2014). These tests tend to correlate with each other, but perhaps not so highly that the tests could be considered interchangeable (Krishnakumar et al. 2016; Mayer et al. 2016).

Goleman (1995) proposed that emotional intelligence would prove to be a strong predictor of workplace success, relationship success, and well-being. We have now conducted enough research to evaluate this proposal. Ability-based assessments of EI have displayed some predictive power with respect to workplace performance (O’Boyle et al. 2011), relationship quality (Lopes et al. 2004), and well-being (Sánchez-Álvarez et al. 2016), but these correlations are often fairly small (around .2) as well as inconsistent (Miners et al. 2018; Roberts et al. 2010). To give some examples, Di Fabio and Kenny (2016) found correlations between the MSCEIT and well-being in the .04 range (very small) and Miao et al. (2017) reported, in a meta-analysis, that ability EI was a weak predictor of workplace citizenship behavior (*r* = .17) and a non-significant predictor of counterproductive work behavior (*r* = .01). Such weak correlations often disappear when controlling for personality and/or cognitive ability (Roberts et al. 2010) and there appears to be uncertainty as to what to do next (e.g., Côté 2014; Matthews et al. 2012; Mayer et al. 2016; Ybarra et al. 2014). Special Issue papers provide relevant answers.

## 2. In Search of Theory

Please see Table 1 for some of the major questions that will be considered in this Special Issue. To begin, much of the knowledge that people have—such as concerning countries in Africa or types of tea—could be considered relatively circumscribed, barely affecting their lives as a whole. People who score high in emotional intelligence presumably have more extensive or accurate knowledge about emotion, yet much of this knowledge may be largely semantic in nature, raising questions about whether or how this knowledge affects the course or tenor of lives, whether in the moment or over longer time frames (Ybarra et al. 2014). Among other points, it should probably be recognized that most behaviors are multiply-determined (e.g., by the situation, by personality, by cognitive abilities) and EI-related influences could be subtle, depending on the situation and/or the behavior (Mayer et al. 2016).

Given such complexities (Mayer et al. 2016), we will simply need to develop some theoretical perspectives on EI, which are surprisingly scarce, in order to understand what these individual differences should predict. Some theorizing could be imported from personality psychology, social psychology, the emotion literature, and/or clinical science. As an example, research on reactive aggression (Wilkowski and Robinson 2010) and emotional impulsivity (Carver et al. 2009) makes the point that emotions often trigger urges to act in an impulsive manner, owing to their links to primitive approach and avoidance systems (Carver et al. 2009; Frijda 2010). People with higher levels of EI, because they possess more extensive knowledge about emotion and its management, may be capable of down-regulating their tendencies toward emotional impulsivity in ways that people with lower levels of EI cannot (Heatherton and Wagner 2011). In support of such theorizing, research has shown that high-EI individuals are less vulnerable to reactive aggression (Gutiérrez-Cobo et al. 2018), distress-influenced suicidal behavior (Cha and Nock 2009), and counterproductive work behavior, particularly when triggered by job negative affect (Krishnakumar et al. 2017).

A related line of work has started to show that individual differences in ability EI, but not self-reported EI, can be linked to cognitive control in emotion-related contexts (Checa and Fernández-Berrocal 2019; Gutiérrez-Cobo et al. 2017). What these process-related abilities would predict in more molar terms is not entirely certain, but these abilities could explain why levels of EI seem to be beneficial to performance under conditions of stress (Udayar et al. 2020). As Udayar et al. (2020) emphasize, retaining control under stressful circumstances could impart a certain sense of self-efficacy in handling emotional arousal, which should render behavior more effective (Bandura 1982). Relatedly, one might expect high-EI individuals to gravitate toward rather than away from emotional stimuli as they would be more confident in their abilities to handle the resulting emotional arousal (Appel et al. 2012).

As suggested by the first two Special Issue papers, another sort of theory is possible. As the materials presented on EI tests require individuals to attach emotional meaning to events or stimuli, it is reasonable to suggest that high-EI individuals (relative to low-EI individuals) are more skilled at doing so. Assuming that similar skills are applied in daily life, the relevant skills should result in higher levels of well-being (e.g., experiences of positive affect) under favorable circumstances, but lower levels of well-being (e.g., experiences of negative affect) under unfavorable circumstances (Cacioppo and Berntson 1999). This framework can explain why high-EI individuals sometimes display higher levels of emotional reactivity in response to stressful events (Bechtoldt and Schneider 2016; Ciarrochi et al. 2002; Matthews et al. 2006). It can also explain the links between EI and well-being (Sánchez-Álvarez et al. 2016), with the presumption that positive events (which would generate higher levels of positive affect among high-EI individuals) tend to be more common than negative events (Alves et al. 2017).

Viewed another way, though, average tendencies (e.g., to report higher or lower levels of well-being in some type of general sense) should not be emphasized. Rather, EI should be associated with dynamic operations (MacCann et al. 2020b), sometimes linking positive evaluations to current conditions (when they are pleasant) and sometimes linking negative evaluations to current conditions (when they are unpleasant). In other words, variations in EI should produce emotional states that are “attuned” to current conditions, as emphasized in functional accounts of emotion (Keltner and Gross 1999). A related construct is psychological flexibility. According to this line of theorizing, psychological health is marked by flexibility, meaning that the person is attuned to situational demands and capable of reconfiguring the self to respond to them (Kashdan and Rottenberg 2010). An important component of psychological flexibility is emotional flexibility, defined in terms of situation-appropriate emotional states (Beshai et al. 2018; Hardy and Segerstrom 2017). Emotional intelligence is very likely to be linked to emotional flexibility, but the implications of this connection are just now being studied (Vanuk et al. 2019).

The point of this section has not been to argue for any particular theory of ability EI. Rather, it has been to argue that we need *some* theorizing, at the present time, so that we can better understand what EI is and what it should do. People with low versus high levels of EI are likely to differ in multiple ways and linking these variations to theory will allow us to make new predictions that can reinvigorate the field.

## 3. Mechanisms and Processes

The “Big Idea” approach contends that emotional intelligence will lead to success in one’s life (Goleman 1995). Given the modest nature of the results that have followed from this perspective (Miners et al. 2018; Ybarra et al. 2014), and given the need for theorizing concerning this class of individual differences, it would seem valuable to identify mechanisms or processes that may represent more proximal, and therefore reliable, correlates. Above, we suggested that emotional intelligence may facilitate processing under emotional circumstances (Checa and Fernández-Berrocal 2019), likely giving rise to a sense of emotion-related self-efficacy, which should benefit self-regulation and performance under stressful circumstances (Schwarzer 2001; Udayar et al. 2020).

Other mechanisms and processes also suggest themselves. When people feel self-efficacious (which we suggest should be linked to higher levels of EI), they are likely to tackle stressful circumstances using a mode of coping termed problem-focused coping (Lazarus and Folkman 1984). This form of coping tends to be adaptive because problems get resolved, clearing a pathway for long-term goal pursuit (Carver and Scheier 2014). In support of this model, MacCann et al. (2011) linked variations in ability EI to variations in problem-focused coping, which in turn predicted higher grade-point averages among students. Results of this type have been replicated (MacCann et al. 2020a) and they suggest that EI is likely to be beneficial in the many circumstances in which problem-focused coping can be advocated (see Carver and Scheier 2014, for a relevant analysis).

Other relevant mechanisms can be found in the psychological flexibility literature. According to this clinical model, human suffering increases as the result of at least two pathological processes (Hayes et al. 2012). Some people are scared of their feelings (i.e., experiential avoidance), which can lead them to restrict their lives in unfortunate manners (Kashdan et al. 2013). Although we are not aware of research linking EI to experiential avoidance, it seems probable that low-EI individuals would be more vulnerable to experiential avoidance given their relative incapacity to understand their feelings. As indicated previously, EI should also be related to emotional flexibility (versus lack of flexibility), defined in terms of situation-appropriate emotional reactions. Following the lead of Klein et al. (2023), high-EI individuals should display intense, but short-lived reactions to both pleasant and unpleasant events.

One can also draw from the emotion regulation literature in making predictions about how EI should operate. According to Gross (2002), emotions can be regulated at various stages of the emotion elicitation process. Much of this research has contrasted to the mechanism of reappraisal, which involves altering appraisals of an eliciting event to alter one’s emotions with suppression, which involves inhibiting the expression of emotions that are felt. Reappraisal can intervene earlier in the emotion eliciting sequence than suppression can, and a considerable body of evidence has pointed to the adaptive nature of reappraisal relative to suppression (John and Gross 2004). There are multiple reasons for thinking that high-EI individuals should be capable of regulating their emotions in more skilled manners (Peña-Sarrionandia et al. 2015), and an emerging body of evidence does in fact suggest positive relationships between ability EI and reappraisal as well as negative relationships between ability EI and suppression (e.g., Megías-Robles et al. 2019; Śmieja et al. 2011). This model can be extended to the correlates of reappraisal and suppression, which include well-being and social behavior (John and Gross 2004).

By regulating negative emotions, high-EI individuals may typically experience lower levels of negative affect (MacCann et al. 2020b). They may also experience higher levels of positive affect, possibly through mechanisms that link EI to engagement with the environment (Robinson et al. 2022). These associations could in turn mediate relationships between variations in ability EI and variations in life satisfaction, eudaimonia, and meaning (Fernández-Berrocal and Extremera 2016). One Special Issue paper explores mediational processes of this type.

## 4. Should We Develop New Tests?

There is an emerging consensus that ability EI consists of three separable, but correlated sets of skills (i.e., “branches”: Mayer and Salovey 1997) that are involved in the perception of emotion, the understanding of emotion, and the management of emotion (Joseph and Newman 2010; MacCann et al. 2014; Shao et al. 2015). But, there is no agreement on the exact materials or scoring procedures that should be used to assess each set of skills. With respect to the perception branch, a number of points have been made. In the MSCEIT (Mayer et al. 2003), perception is assessed with two tasks, one of which involves identifying emotions in faces and the other of which involves identifying emotions in pictures (e.g., abstract paintings). The former task is probably more central to emotion perception than the latter (Hall et al. 2009) and interventions designed to increase emotion perception have succeeded in altering face perception, but not picture perception (Herpertz et al. 2016). Hence, there are doubts about the picture perception task.

Face perception tasks often use high-intensity, prototypical expressions. In daily life, however, emotional displays tend to be less intense and less prototypical (Matsumoto and Hwang 2014). There could be value in assessing decoding ability with respect to less intense stimuli, which might capture skills that are more often used in daily interactions with others (Matsumoto and Hwang 2014). In addition, some EI experts have contended that static facial expressions are limited and have called for assessments of emotion perception accuracy in relation to more dynamic materials such as videos (Schlegel et al. 2014).

Also pertaining to ecological validity, some theorists have emphasized the importance of context in the manner in which emotion perception processes operate (Barrett et al. 2011). The idea here is that facial perception in particular, as well as person perception more broadly, rarely occurs in a context-free manner, such that many sources of contextual meaning impact the perceptions that people have (also see van Kleef and Côté 2022). Some of these contextual features of meaning can be added to emotion perception materials. Hess and Kafetsios (2021) have explored procedures of this type by presenting target expressers together with surrounding expressers (i.e., other individuals). Procedures of this type can allow one to calculate separable measures of accuracy (perceiving the intended emotions) and bias (perceiving additional emotions to those intended), with each type of score possessing social cognition significance. One of the Special Issue papers reviews this research program.

In the MSCEIT, emotional understanding (EU) is assessed by asking test-takers about combinations of emotion that are likely to be felt by target characters. But, emotional understanding encompasses a broad set of processes (Castro et al. 2016) and other assessment procedures could be used. Hellwig and Schulze (2021) describe a new EU test that incorporates appraisal information into situational descriptions. A good test-taker is able to infer the likely emotions that would be felt on the basis of the appraisals that were made, resulting in a theory-informed scoring system (also see MacCann and Roberts 2008). Another particularly ambitious test describes situations that would likely give rise to 1 of 10 emotions. With respect to each scenario, test-takers make inferences concerning the appraisals, action tendencies, expressions, and subjective feelings of each character (Sekwena and Fontaine 2018). The skills involved in these inferences are numerous and the test is, therefore, a particularly comprehensive one. As readers will encounter, one of the Special Issue papers also discusses the creation of EU tests based on core relational themes (i.e., molar summaries of the appraisals linked to a particular emotion: Smith and Lazarus 1993).

The management branch of the MSCEIT seems to capture important intrapersonal and interpersonal skills (Lopes et al. 2004; MacCann et al. 2011), but there are questions concerning the assessment of these skills. The skills involved in managing one’s own emotions, for example, are probably different than the skills involved in managing the emotions of others and these skills might be distinguished (Durham et al. 2023). The emotion regulation literature has also made a great deal of progress in understanding the different types of emotion regulation strategies that people can use (e.g., Olderbak et al. 2023), but these developments have not been incorporated into ability EI tests in any systematic manner. Finally, the emotion regulation literature has increasingly suggested that people regulate their emotions for instrumental as well as hedonic reasons (Tamir 2016) and the former sorts of reasons could be modeled in ability EI tests to a greater extent (also see Mayer et al. 2016 for additional thoughts about expanding the emotion management testing space).

In summary, EI researchers are busy developing new tests of ability EI, some of which are discussed in this Special Issue. It is uncertain whether some of the tests could be packaged together such that there is a new comprehensive test like the MSCEIT. If not, we will at least have new tests targeting particular branches that are likely to display higher levels of predictive validity. With respect to this point, one last development should be mentioned. Organizational researchers have found that altering generic personality measures such that they target a particular context (e.g., the workplace) results in higher-validity coefficients when predicting outcomes pertinent to that context (Bowling and Burns 2010; Shaffer and Postlethwaite 2012). Results of this type have inspired ability EI tests targeting the workplace (Krishnakumar et al. 2016; Schlegel and Mortillaro 2019) and one could imagine similar developments targeting other contexts (e.g., interpersonal relationships: Pratscher et al. 2019).

## 5. Connecting with the Other Literature

As Hoemann et al. (2021) emphasize, much of the literature has proposed variations in emotion-related expertise, with relevant constructs including alexithymia, emotional awareness, emotional clarity, emotional complexity, emotional competence, empathic accuracy, and emotional intelligence. Although the test procedures used to assess ability EI may be somewhat unique, it would be surprising if there were no links (whether empirical or theoretical) between emotional intelligence and other expertise-related constructs that have been proposed. Empathic accuracy, which conceptually overlaps with the perception branch of EI (Mayer and Salovey 1997), quantifies the extent to which inferences concerning the thoughts and feelings of a target, typically after a communication episode, overlap with the actual thoughts and feelings of the target, as reported on by the target (Hall and Schmid Mast 2007). Such skills seem to be highly dependent on who the target is and whether the perceiver is motivated to understand the thoughts and feelings of that target in a particular setting (Sassenrath et al. 2022). Aside from this point, this piece of literature is useful in highlighting several potential downsides to empathic accuracy, such as the possibility that these skills can be used for Machiavellian purposes (Hodges and Myers 2007) and that they can threaten relationships, such as when a person correctly infers that their partner is attracted to another person (Simpson et al. 2003). The potential downsides to emotional intelligence, thus far, have only received scattered attention (Davis and Nichols 2016).

Emotional awareness encompasses two constructs—attention to emotion and emotional clarity. Some people value their emotions to a greater extent and they attend to them for this reason. Such individuals tend to report stronger reactions to emotional events, but they also display emotional wisdom (e.g., by choosing to avoid events that would give rise to negative emotion: Robinson et al. 2021). Emotional clarity is meta-cognitive in nature and it occurs when people sense that they understand their emotions well (Boden and Thompson 2017). Like emotional intelligence, there are individual differences in emotional clarity and they are associated with higher levels of well-being (Lischetzke et al. 2012) as well as lower levels of psychopathology (Vine and Aldao 2014). But emotional clarity also varies quite a bit within a person, with predictable consequences (Thompson and Boden 2019). The ability EI literature has not yet focused on within-person changes in EI, with the exception of intervention studies (Durham et al. 2023). Theorizing at this within-person level might allow researchers to better understand the “fluid” (Fiori et al. 2022) aspects of EI.

Emotional intelligence should, ideally, support achievements such as wisdom, maturity, and resilience. These are difficult constructs to measure, but our understanding of optimal functioning requires such efforts (Seligman and Csikszentmihalyi 2000). Resilience seems to involve some paradoxical elements. On the one hand, resilient people are more reactive to pleasant and unpleasant events in momentary experience (Waugh et al. 2011). On the other hand, resilient people are capable of experiencing positive emotions under adverse circumstances (Fredrickson et al. 2003) and they are capable of rebounding from negative circumstances more quickly (Masten 2001). In other words, resilience seems to promote “stability through change”, which is a fundamental feature of healthy physiological and psychological systems (McEwen and Lasley 2002). Resilience clearly involves abilities and these abilities clearly involve appraisals and emotions (Tugade and Fredrickson 2004). In future research, it would seem valuable to attempt to operationalize such skills in ability-related terms.

## 6. Can Emotional Intelligence Be Trained?

One of the benefits of the ability EI model is that it conceptualizes EI in terms of skills that could, potentially, be trained (Hoffmann et al. 2020). We now have enough of this research that it is safe to conclude that EI can be trained, although effect sizes are medium rather than large (Hodzic et al. 2018). The literature has limitations, however. Among them, Kotsou et al. (2019) suggest the need for more studies that randomly assign participants to intervention versus control groups, that use “active” control groups to guard against expectancy effects, that use ability EI measures as outcomes, and that examine long-term as well as short-term changes. There is also the need to create standardized interventions, with a clearly specifiable content, which will facilitate comparisons among training procedures in future efforts (Kotsou et al. 2019). In addition to these developments, one Special Issue paper considers the question of whether EI training is efficacious when it is delivered online rather than in face-to-face terms. Success with such digital interventions will permit wider dissemination, though one issue is that volunteers in such studies will tend to have higher levels of education as well as (quite likely) higher levels of pre-existing EI, which would be linked to interest in volunteering for EI studies.

## 7. Additional Future Directions

As we reflect on the state of the ability EI literature, many questions suggest themselves. One question is whether ability EI can be linked to interoceptive abilities, defined in terms of individual differences in the accurate representation of afferent signals from the body (Herbert and Pollatos 2012). Bodily signals clearly contribute to emotional experience (Critchley and Garfinkel 2017), but EI tests also seem to assess variations in semantic knowledge concerning emotion, which would not vary as a function of current bodily experiences (Hoemann et al. 2021). Nonetheless, data do suggest that interoceptive abilities are involved in the representation (Zaki et al. 2012) and experience (Dunn et al. 2010) of emotion, such that links between ability EI and interoception might be expected (also see Klein and Robinson 2021). Given these potential links, as well as their importance to theories of emotion (Zaki et al. 2012), further work on this EI–interoception interface seems warranted.

One conception of emotional expertise contends that it involves differentiated feelings, which can be assessed by computing within-subject correlations between emotional experiences of a given valence (e.g., the anger–sadness correlation across reports of experience, with lower correlations suggesting a greater differentiation of these emotions: Smidt and Suvak 2015). Briefly, it is thought that higher levels of emotion differentiation support behavior that is more emotionally intelligent (Kashdan et al. 2015). Ability EI tests ask people to make distinctions among emotions (e.g., anger versus sadness) and it is intuitive to suggest that individuals obtaining high EI scores will exhibit greater emotion differentiation in their daily lives. However, MacCann et al. (2020b) report results that are contrary to this prediction. Hence, it would seem that more work is necessary in clarifying the relationship between ability EI and emotion differentiation. If emotion differentiation is not captured by current tests, we may need to create new ones.

More generally, we need to know a lot more about whether and how ability EI manifests itself in daily life. With respect to this point, the emotion dynamics literature has used variations of the experience-sampling paradigm (Scollon et al. 2003) to answer many questions about variations in emotional reactivity, emotion regulation, and other components of emotion change (e.g., inertia: Koval et al. 2015; see Kuppens et al. 2022, for a general review). In the future, we would like to see a greater integration of the EI and the emotion dynamics literature, whether in relation to laboratory (Klein et al. 2023; Waugh et al. 2011) or experience-sampling (MacCann et al. 2020b) paradigms. As shown in some examples, high-EI individuals may exhibit stronger emotional reactions to daily events of a given valence (Beshai et al. 2018), but they may be more capable of regulating pathological reactions to such events (Robinson et al. 2012). Clearly, there are complexities here that merit research.

A prominent neuroscience model contends that damage to emotion representation regions of the brain severely impairs everyday decision making (Naqvi et al. 2006). Such patients not only display flat affect, but they have trouble making life choices and they take unwarranted risks (Bechara 2004). Since such patients have preserved cognitive abilities, this research highlights the functional importance of feelings in decision making (Naqvi et al. 2006). Low levels of ability EI may act in a manner akin to this neurocognitive model, but very little research has focused on this possibility. Among other predictions, low-EI individuals may perform more poorly in tasks such as the Iowa Gambling Task (Bechara et al. 2000) and they may display some degree of insensitivity to hedonic considerations in tasks such as those described by Caruso and Shafir (2006) or Robinson et al. (2021).

As stated above, it is recognized that there are distinct sets of EI skills that can be broadly grouped into the perception, understanding, and management areas (Joseph and Newman 2010). Although these skill sets are separable, they tend to load onto a global EI factor (Krishnakumar et al. 2016; MacCann et al. 2014). In the future, it seems likely that some researchers will prefer to theorize at the branch level (e.g., He and Côté 2023), while others will prefer to theorize at the global level (e.g., Robinson et al. 2019). It is not clear which level of theorizing is best, but the development of new tests pertaining to particular branches (e.g., Elfenbein et al. 2017; Sekwena and Fontaine 2018) will likely fractionate the literature to a certain extent. This trend could be countered by focusing on ways in which different EI skill sets interact with each other, much as mindfulness-related skills do (Eisenlohr-Moul et al. 2012).

## 8. Conclusions

In this introduction to a Special Issue on ability EI, we emphasized the importance of developing new theories, of focusing on mechanisms and processes, of developing new EI tests, of connecting with the other literature, and of training studies that answer novel questions. As displayed in Table 2, the Special Issue papers cover all of these topics. Emotional intelligence should matter in diverse realms such as decision making, social behavior, and well-being, but new developments are needed to forge these links. The present Special Issue is a timely one and it is hoped that we will learn considerably more about what ability EI is and how it works in upcoming years.

## Figures and Tables

**Table 1:** Some Questions Asked in the Special Issue (and/or Introduction).

Focus	Questions
Theory	How does emotional knowledge impact people’s lives?
	Are low-EI individuals prone to emotionally impulsive behaviors?
	How would cognitive control in emotional contexts manifest itself?
	Might EI be linked to higher levels of emotional reactivity?
	Is EI linked to psychological flexibility?
Mechanisms	Do high-EI individuals favor problem-focused coping?
	Might low-EI individuals be prone to experiential avoidance?
	Do high-EI individuals reappraise rather than suppress their emotions?
	Is EI linked to average levels of positive and negative affect?
Measurement	Should emotion perception materials use fewer prototypical stimuli?
	How can we model social context in emotion perception tasks?
	Can we develop theory-informed tests of emotional understanding?
	Can emotion regulation theories inform emotion management assessments?
	Should we develop more context-specific assessments of EI?
Neighboring Areas	Are higher perception abilities always beneficial?
	Might EI levels display within-person fluctuations?
	Can EI be linked to wisdom, maturity, or resilience?
	Can we develop ability-based approaches to resilience?
Training	Can emotional intelligence be trained?
	What sorts of skills should be trained in intervention studies?
	Can EI be trained using online training methods?
Future Directions	Do interoception abilities contribute to emotional intelligence?
	Do high-EI individuals experience emotions in a differentiated manner?
	What are the daily diary signatures of ability EI?
	How does ability EI impact decision making?
	Do different EI skills interact with each other?

Note: EI = Emotional Intelligence.

**Table 2:** An Overview of Special Issue Papers.

Focus	Number	Brief Summary
Theory	1	Presents a hypersensitivity theory of EI
	2	Presents an evaluation expertise theory of EI
Mechanisms	3	Positive and negative affect as well-being mediators
Measurement	4	A contextual approach to emotion perception
	5	Integrating emotion theory into EI measurement
	6	A new test of emotion perception
	7	A new test of emotional understanding
Neighboring Areas	8	A review of the correlates of empathic accuracy
	9	Emotional clarity as fluid EI
	10	Resilience from skill and well-being perspectives
Training	11	An online intervention for emotional intelligence

Note: EI = Emotional Intelligence.

## Data Availability

Not Applicable.
